# Immune lymphocytes halt replication of *Francisella tularensis* LVS within the cytoplasm of infected macrophages

**DOI:** 10.1038/s41598-020-68798-2

**Published:** 2020-07-21

**Authors:** Mary Katherine Bradford, Karen L. Elkins

**Affiliations:** 1 Laboratory of Mucosal Pathogens and Cellular Immunology, Division of Bacterial, Parasitic and Allergenic Products, Center for Biologics Evaluation and Research, U.S. Food and Drug Administration, Silver Spring, MD 20993 USA; 2 Present Address: Johns Hopkins University Professional Development and Career Office, 1830 E. Monument, 2-107, Baltimore, MD 21287 USA

**Keywords:** Bacteria, Bacteriology, Pathogens, Vaccines, Experimental models of disease, Adaptive immunity, Antimicrobial responses, Imaging the immune system, Infection, Infectious diseases, Lymphocytes, Vaccines

## Abstract

*Francisella tularensis* is a highly infectious intracellular bacterium that causes tularemia by invading and replicating in mammalian myeloid cells. *Francisella* primarily invades host macrophages, where it escapes phagosomes within a few hours and replicates in the cytoplasm. Less is known about how *Francisella* traffics within macrophages or exits into the extracellular environment for further infection. Immune T lymphocytes control the replication of *Francisella* within macrophages in vitro by a variety of mechanisms, but nothing is known about intracellular bacterial trafficking in the face of such immune pressure. Here we used a murine model of infection with a *Francisella* attenuated live vaccine strain (LVS), which is under study as a human vaccine, to evaluate the hypothesis that immune T cells control intramacrophage bacterial growth by re-directing bacteria into toxic intracellular compartments of infected macrophages. We visualized the interactions of lymphocytes and LVS-infected macrophages using confocal microscopy and characterized LVS intramacrophage trafficking when co-cultured with immune lymphocytes. We focused on the late stages of infection after bacteria escape from phagosomes, through bacterial replication and the death of macrophages. We found that the majority of LVS remained cytosolic in the absence of immune pressure, eventually resulting in macrophage death. In contrast, co-culture of LVS-infected macrophages with LVS-immune lymphocytes halted LVS replication and inhibited the spread of LVS infection between macrophages, but bacteria did not return to vacuoles such as lysosomes or autophagosomes and macrophages did not die. Therefore, immune lymphocytes directly limit intracellular bacterial replication within the cytoplasm of infected macrophages.

## Introduction

*Francisella tularensis* is a gram-negative, facultative intracellular bacterium that replicates in macrophages and causes tularemia in humans^1–3^. *Francisella* can infect people via multiple routes, but respiratory infection leads to the most severe form of the disease and can be fatal if not treated. *F. tularensis* is found throughout North America and is endemic in Europe, especially in Scandinavia, and in Asia. Tularemia is not a large public health concern in developed countries. However, *Francisella* was investigated as a bioweapon in the mid-1900s by both the United States and Soviet Union; the bacterium is therefore is currently categorized as a Select Agent in the United States^3^.

The intracellular lifecycle of *Francisella* has been visualized in vitro with fixed and live-cell microscopy. *Francisella* enters macrophages via phagocytosis and escapes from the resulting phagosome within 1 to 4 h, avoiding lysosomal fusion^4–13^. Cytosolic *Francisella* replicates to high numbers over the next day^9^. To date, in-depth characterization has focused on the first 24 h after infection of host myeloid cells. One set of reports has described clustering of LVS about 20 h after infection of murine macrophages into “*Francisella*-containing vacuoles” (FCVs), which are distinct from the initial bacterial entry phagosomes^9,14^. However, the formation of FCVs appears to be specific to LVS infection of murine cells, and FCVs do not appear in infection of human cells with other strains of *Francisella*^15–17^. Thus, the prevalence, function, and significance of FCVs remains unclear. Further, intracellular infection and trafficking of LVS in the face of immune pressure from lymphocytes has not yet been directly visualized or characterized.

Currently, no vaccines are licensed against tularemia in the United States. The most well-studied *Francisella* vaccine is an attenuated strain denoted Live Vaccine Strain^2,18–20^. Infection of mice with LVS can vaccinate animals or cause tularemia-like symptoms and death depending on route of infection, making LVS a useful BSL-2 model of infection^19,21,22^. We have used this model to study mechanisms of protective immunity against intracellular pathogens in general and *Francisella* in particular, and to evaluate vaccines and correlates of vaccine-induced protection. To dissect mechanisms, we developed an in vitro co-culture assay in which primary bone-marrow derived macrophages (BMM) are grown in monolayers, infected with LVS or other *Francisella* strains, and lymphocytes from either naïve or vaccinated mice are overlaid on infected macrophages. The resulting interactions are evaluated in terms of impact on intramacrophage bacterial replication and immune responses, including gene expression and mediator production^23,24^. In this setting, *Francisella* invades macrophages and replicates to high levels either without lymphocytes or when naïve lymphocytes are overlaid, as assessed by bacterial colony-forming units (CFUs). In contrast, lymphocytes from LVS-vaccinated mice strongly limit intramacrophage LVS replication.

The mechanisms of in vitro bacterial growth control depend on CD4^+^ or CD8^+^ effector T cell functions, including IFN-γ, TNF-α, and nitric oxide (NO) production, as well as roles for IL-6, T-bet, and IL-12Rβ2; in contrast, B cells, NK cells, and myeloid cells have minimal if any contributions^23–29^. In mice and rats, this in vitro co-culture system has proved to provide a functional correlate of vaccine-induced protection in vivo^30–34^, supporting its relevance for studies of infection and in vivo mechanisms central to protective immunity. Results indicate that mechanisms identified to date do not account for all bacterial growth control, and thus additional mechanisms by which T cells limit intramacrophage bacterial growth await discovery^24,25,35^.

Here, we evaluated another potential T cell effector mechanism. We hypothesized that LVS-primed lymphocytes may re-route intracellular trafficking of LVS into compartments equipped to kill bacteria. We adapted the in vitro co-culture approach to directly evaluate the effects of LVS-immune lymphocytes on intracellular trafficking of *Francisella* in mouse macrophages. We demonstrate that LVS infection of macrophages resulted in extensive bacterial replication and eventually death of infected macrophages, although cell death processes were quite heterogeneous. Most importantly, lymphocytes from LVS-vaccinated mice, but not naïve mice, strongly inhibited intercellular bacterial spread and intracellular bacterial replication within the cytoplasm of infected cells, with minimal re-routing of bacteria to lysosomal, endosomal, or autophagic vesicles.

## Results

### Intracellular growth and intercellular spread of LVS in in vitro BMM cultures

**Figure 1:**
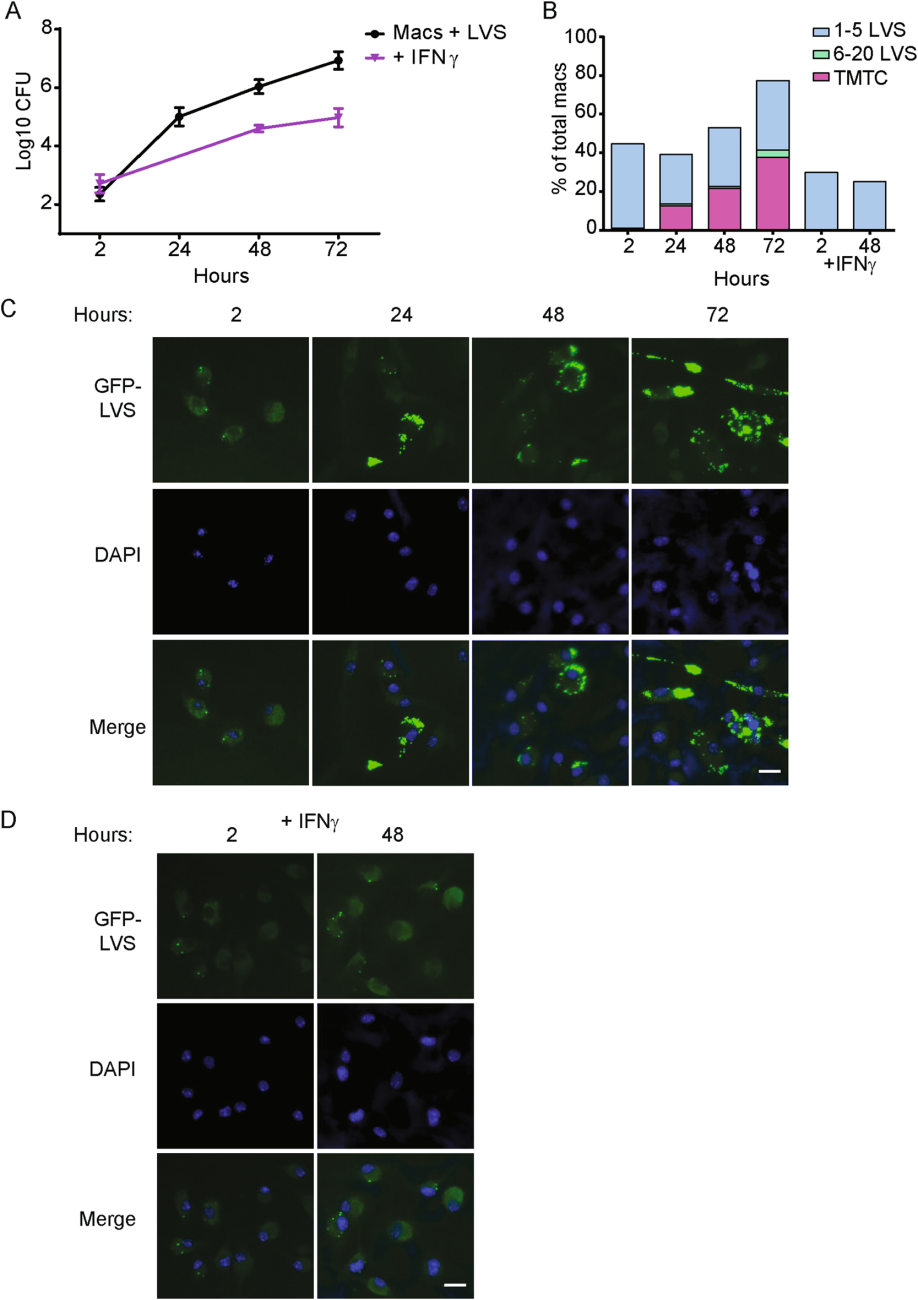
In vitro intracellular growth and intercellular spread of LVS in mouse BMM. Murine BMM were cultured without or with 50 ng/ml recombinant mouse IFN-γ for 24 h, as indicated, and then infected with GFP-LVS at an MOI of 50:1 (bacteria to macrophage ratio). (**A**) At the indicated time points after infection, macrophages were washed, lysed, and plated to evaluate the recovery of intracellular bacteria. Values shown are the mean numbers of CFU/ml of viable bacteria ± SD of triplicate samples. (**B**) GFP-LVS-infected macrophages were scored by visual inspection for numbers of bacteria in each macrophage. The level of infection was categorized into four groups: macrophages containing no bacteria, 1–5 bacteria, 6–20 bacteria, or > 21 bacteria as too many to count (TMTC). At least 100 macrophages from two identical replicates were scored for each condition. (**C**, **D**) Representative images of BMM infected with GFP-LVS ± IFN-γ at indicated time points after infection. Scale bar = 50 µM. Results shown are from one representative experiment of three independent experiments of similar design and outcome.

**Figure 2:**
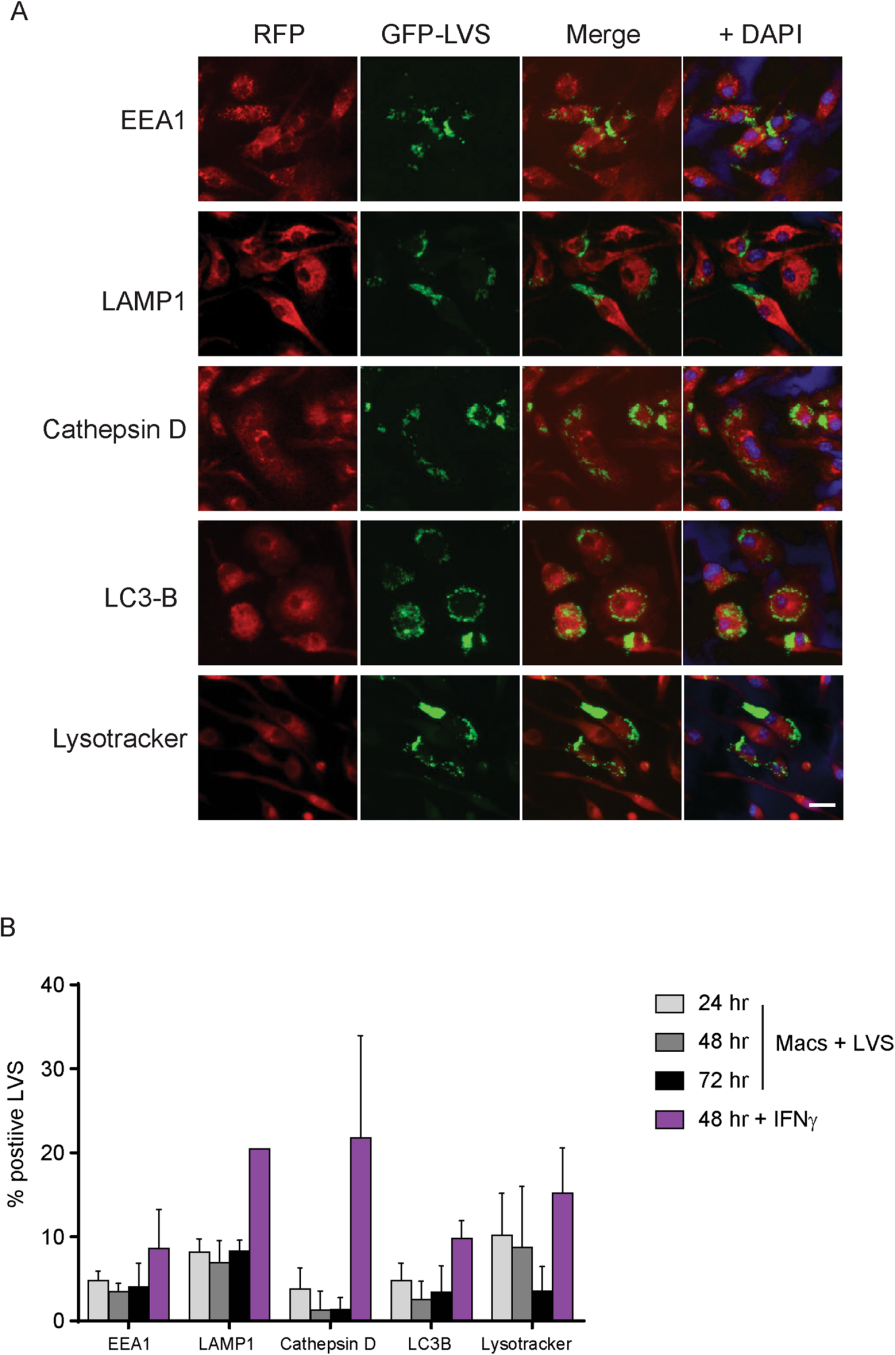
GFP-LVS replicates to high numbers in murine BMM, and LVS remains cytosolic but not associated with vacuoles. Murine BMM were cultured ± 50 ng/ml recombinant mouse IFN-γ for 24 h, as indicated, and then infected with GFP-LVS at an MOI of 50:1. (**A**) Representative images of GFP-LVS-infected (green) macrophages (not treated with IFN-γ) with the indicated trafficking markers (red) 48 h after infection. Macrophage nuclei were stained with DAPI (blue). Scale bar = 50 µM. (**B**) Colocalization of single GFP-LVS bacteria with markers, as illustrated in (**A**), was quantified at the indicated time points after infection. At least 70 bacteria were scored from two identical replicates for each condition. Results shown are combined means ± SD for three independent experiments. No significant (*P* > 0.05) or consistent differences were found between any combination of groups.

We first adapted and optimized the in vitro co-culture assay for visualization by combining previous protocols^9,23,24^ to investigate the intramacrophage trafficking of LVS under immune pressure from LVS-immune lymphocytes. To allow optimal visualization with sufficient sensitivity and resolution, we increased the multiplicity of infection (MOI) to 50:1 (bacteria:macrophage), synchronized the infection initiation, decreased the density of macrophages, and cultured the samples on coverslips (data not shown). Of note, at the time of infection, bone marrow-derived macrophages used here were terminally differentiated and exhibited little if any further cell growth during the culture period (data not shown). Using these revised conditions, we first characterized the later stages of synchronous LVS infection of macrophages between 24 and 72 h; we included LVS-infected macrophages treated with IFN-γ as a comparator. Consistent with previous reports, LVS tagged with a fluorescent reporter (GFP-LVS or mCherry-LVS) was phagocytosed by macrophages and began to escape from EEA1^+^ or LAMP1^+^ phagosomes within 1 to 2 h of infection (Supplementary Fig. 1, illustrating partial LVS colocalization with LAMP1 by 4 h after infection). By 24 h after infection, most LVS was no longer associated with LAMP1 (Supplementary Fig. 2), nor was not associated with EEA1, cathepsin D, or Lysotracker, or LC3-B (data not shown, and see Fig. 2). In contrast, LVS-ΔpdpA, a mutant lacking a *Francisella* pathogenicity island protein that restricts phagosomal escape^36^, remained associated with LAMP1 as previous reported (Supplementary Fig. 2). Following phagosomal escape, wild type LVS replicated to high numbers. LVS growth was significantly reduced (but not halted) in macrophages that were pre-activated with IFN-γ (Fig. 1A). Numbers of GFP-LVS in each macrophage, as well as numbers of infected macrophages, were further quantified using fixed microscopy. Four categories of macrophages were identified based on the number of GFP-LVS present: zero bacterium, one to five bacteria, six to twenty bacteria, or > twenty-one bacteria (too many to count, TMTC). Within the first 24 h after infection, the overall percentage of infected macrophages remained about 40%, but the number of LVS per macrophage increased, and by 24 h about 15% of macrophages contained greater than 20 bacteria (Fig. 1B). Between 48 and 72 h after infection, the percentage of infected macrophages increased from 50 to 80% and the number of LVS per macrophage also continued to increase, indicating that LVS was spreading between macrophages in the culture. Representative images illustrate the increase in GFP-LVS over time in culture (Fig. 1C). In contrast, in macrophages that were pre-activated with IFN-γ, only about 30% of macrophages were initially infected, and LVS did not appreciably replicate nor spread to neighboring macrophages (Fig. 1B, D). Thus, visualization and quantification of LVS infection of mouse macrophages over 3 days confirms that the infection spreads to 80–90% of macrophages, with nearly half of the macrophages harboring large numbers of bacteria.

### LVS remains cytosolic throughout mouse macrophage infection

Depending on cell types and *Francisella* strains, intracellular *Francisella* has been reported to re-enter autophagic-like vacuoles after phagosomal escape and continue replicating^9,15–17^. We therefore evaluated the intracellular localization of both single LVS bacteria and LVS masses, defined as large aggregates of LVS grouped together such that individual LVS bacteria could not be distinguished. We focused on mouse macrophages between 24 and 74 h after infection, using confocal microscopy coupled with staining for markers for the endosomal (EEA1 and LAMP1), autophagic (LC3B), and lysosomal (Cathepsin D and Lysotracker) pathways. Representative images of LVS infection in association with these markers are shown in Fig. 2A, and GFP-LVS colocalization is quantified in Fig. 2B. In contrast to previous reports, we found that greater than 90% of single LVS bacteria remained cytosolic for all 3 days of infection (Fig. 2A, B). Addition of IFN-γ slightly increased the colocalization of LVS with markers of the autophagosomal as well as lysosomal pathways, but differences were not significant nor consistent across experiments (Fig. 2B).

In separate experiments, we also evaluated LVS localization using a previously described phagosomal integrity technique that relies on differential staining of bacteria in cytoplasm compared to those within membrane-bound vacuoles^9^. These studies confirmed that LVS began to escape from vacuoles within 1 h after infection but was largely cytosolic at 24 h and not sequestered in vacuoles (Supplementary Fig. 3). In this example, 84% of LVS bacteria were scored as cytoplasmic at 24 h; scoring at 48 and 72 h was not considered reliable due to increasing fragility of the cells (data not shown). Further, intracellular bodies that resembled the previously-described FCVs^9^ in macrophages were very rarely seen in studies using differential permeabilization. Similarly, rare bodies similar to FCVs were seen in studies that used marker staining to evaluate the localization of LVS within cells, such as staining for LAMP1, as previously reported^9^ (Supplementary Fig. 4). Across experiments using either differential permeabilization or marker staining, less than 1% of 1,300 macrophages containing greater than 20 bacteria contained an FCV-like body (data not shown), and thus FCVs were very unusual under the conditions used here.

### Macrophages die during course of LVS infection

**Figure 3:**
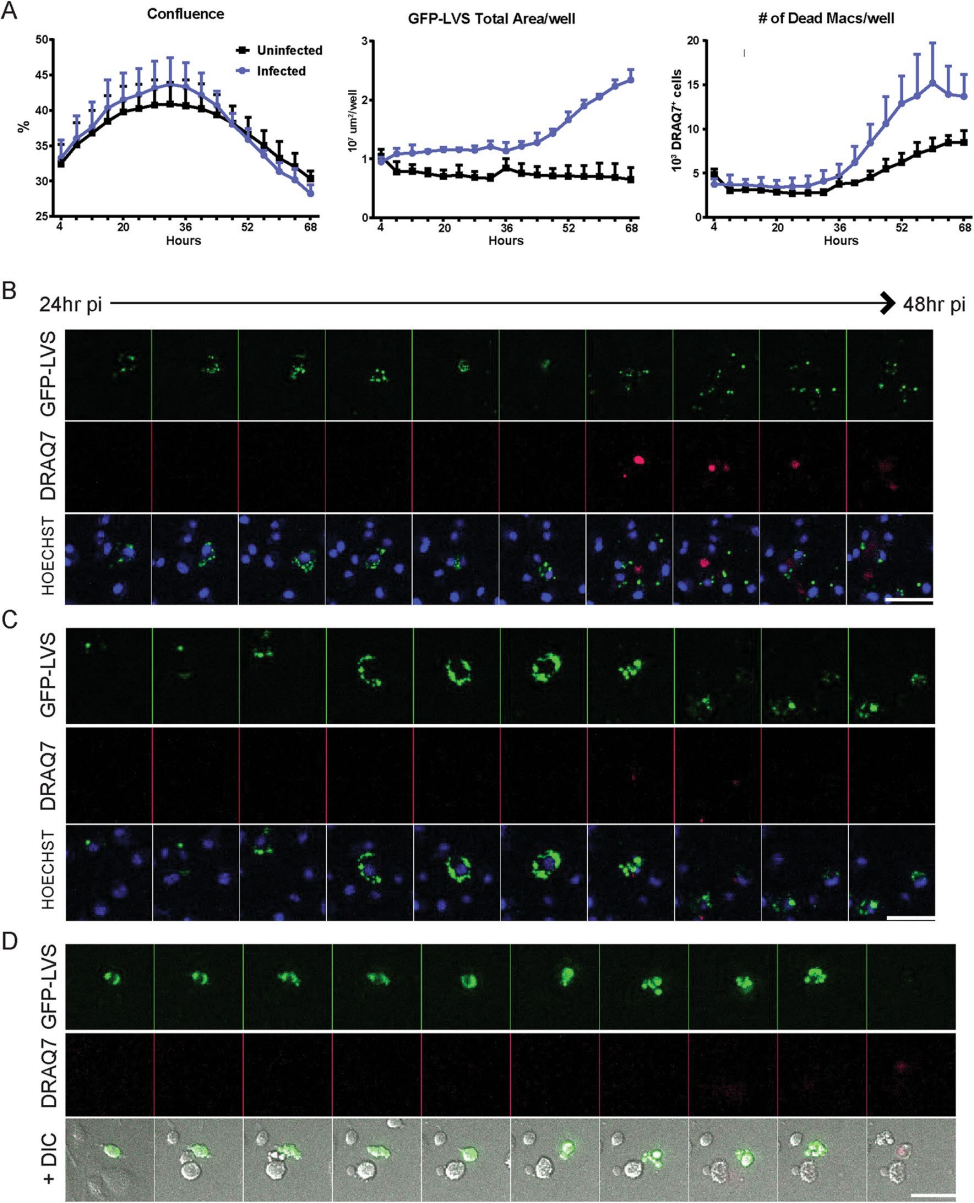
GFP-LVS-infected macrophages die as LVS replicates and spreads within cultures. Murine BMM were infected with GFP-LVS at an MOI of 50:1 (bacteria to macrophage ratio). (**A**) An Incucyte ZOOM was used to automatically acquire whole-well, phase contrast, GFP (LVS) and RFP (DRAQ7) images of GFP-LVS-infected BMM in 24 well plates every 4–6 h. Incucyte’s automated image analysis software was used to determine percent confluency (left panel), total GFP area per well (middle panel), and the number of DRAQ7^+^ cells per well (right panel). Triplicate wells were averaged, and results shown are mean +/– SD for one representative experiment of two independent experiments. (**B**–**D**) Between 24 and 48 h after infection, GFP-LVS infected macrophages (green) cultured in chambered coverslips were imaged with spinning disk microscope every 10 (**D**) to 15 (**B**, **C**) minutes for 24 h. Time lapse movies (Supplementary Movies 1–3) representative of three distinct patterns of cell death were compiled, and montages of static images of representative infected macrophages are shown. DRAQ7 (red) was added to all wells prior to imaging to label dead and dying cells. (**B**, **C**) Hoechst dye was used to label the macrophage nuclei. (**D**) DIC was imaged to visualize cell membranes. The bottom panels in (**B**)–(**D**) provide merged images with all three parameters illustrated. Scale bars are 50 µM. Results shown are representative of images observed in three independent experiments.

In addition to characterizing the intracellular localization of LVS, the in vitro co-culture approach also allows for direct observation of the fate of infected macrophages throughout the 3-day infection. Death of *Francisella*-infected macrophages has been studied previously using traditional population-based assays^37–42^. Here, we extended previous work by visualizing this dynamic process in live individual cells through late time points of infection. We used an Incucyte ZOOM instrument to measure GFP-LVS replication in the context of macrophage confluence and cell death, and we used spinning-disk microscopy to visualize LVS-infected macrophage morphology and dissolution at the single cell level. Within the first 36 h of culture, macrophage confluency was stable and then declined between 36 and 68 h, regardless of LVS infection (Fig. 3A, left panel). However, especially in infected cultures, macrophage death (as reflected by increased DRAQ7 uptake; Fig. 3A, right panel) increased between 36 and 68 h, in parallel with exponential LVS replication (as reflected by the increase of GFP fluorescence over time; Fig. 3A, middle panel).

To visualize how LVS escapes from macrophages, GFP-LVS-infected macrophages were imaged by spinning disk microscopy every 10–15 min for 24-h time periods during the 3-day infection. Hoechst dye was added to stain nucleic acid, making macrophages easily discernable; DIC images were taken to observe macrophage cell membranes; and DRAQ7 dye was added to indicate a decrease in macrophage membrane integrity and macrophage death. Many phenotypes of LVS replication and macrophage death were observed, indicating a variety of dynamic processes. Three representative examples of infected macrophage death phenotypes are illustrated in the montages in Fig. 3B–D, which correspond to the time lapse movies in Supplementary Movies 1–3. Some LVS-infected macrophages were motile within the culture wells and then became stationary as their membranes blebbed, swelled, or ruptured. In Fig. 3B (Supplementary Movie 1), GFP-LVS replicated until the macrophage’s membrane became compromised and the nucleic acid became DRAQ7^+^; GFP-LVS then appeared to spread to neighboring macrophages. As illustrated in Fig. 3C (Supplementary Movie 2), GFP-LVS replicated to high numbers and filled the cytosol, then re-localized to the perimeter of the macrophage as the cell rounded; just before membrane rupture, GFP-LVS appeared to localize into discrete aggregates. Released LVS was phagocytosed by neighboring macrophages, but it was not clear whether this LVS was free in the extracellular environment or was packaged within macrophage membrane blebs prior to being phagocytosed. In the final representative time lapse shown in Fig. 3D (Supplementary Movie 3), the macrophage membrane was visualized in the DIC channel (bottom panel). The GFP-LVS-infected macrophage formed a number of dynamic membrane blebs for a few hours, then the membrane was compromised and the cell became DRAQ7^+^. After membrane compromise, GFP-LVS appeared to spill into the extracellular environment unaggregated.

### Macrophages phagocytose splenocytes during in vitro co-culture assays

**Figure 4:**
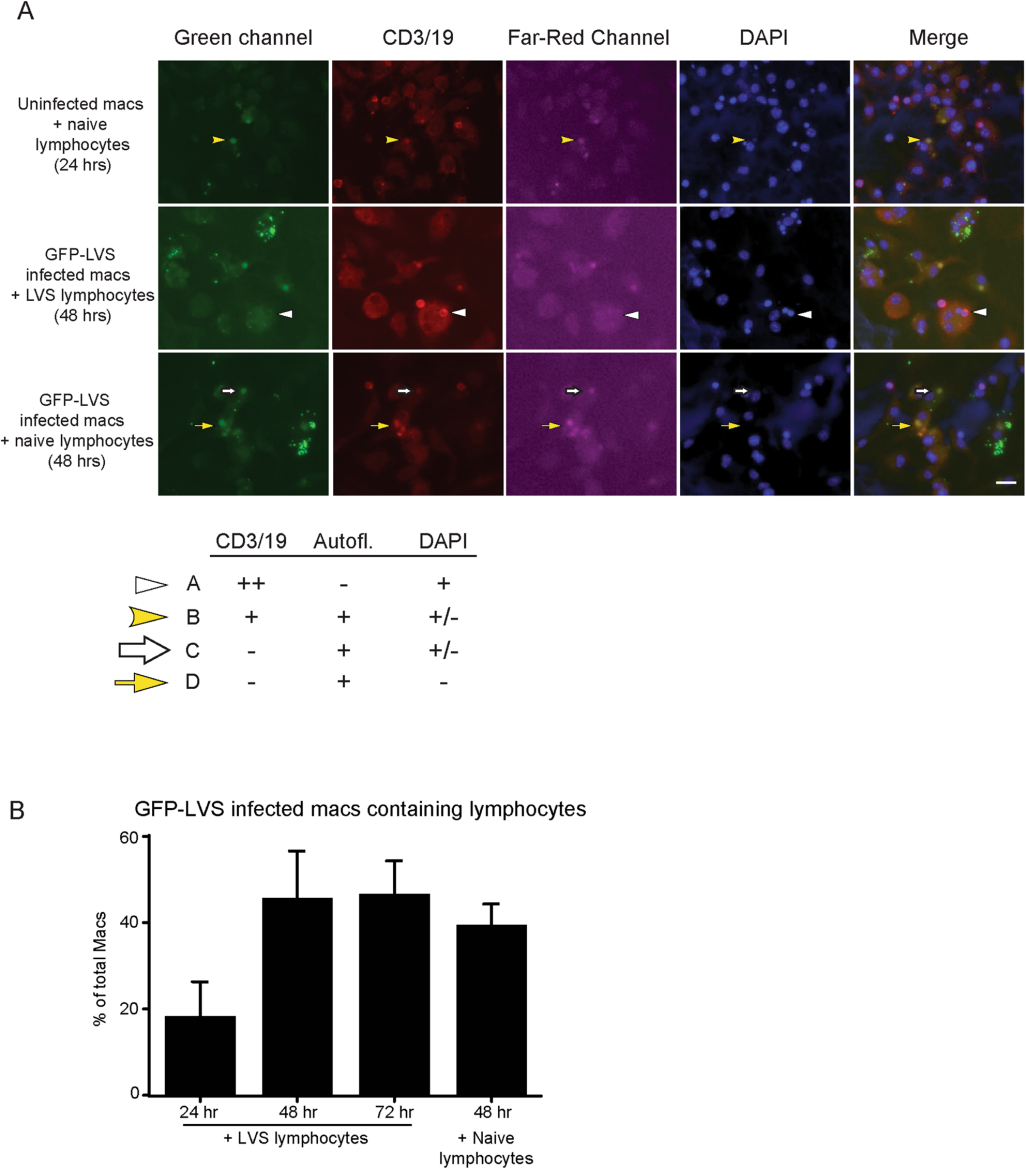
Macrophages phagocytose lymphocytes during in vitro co-cultures. GFP-LVS-infected or uninfected murine BMM were co-cultured with splenic lymphocytes from LVS-vaccinated or naïve mice for 24–72 h, as indicated. (**A**) Representative confocal microscopy images are shown. The green channel depicts GFP-LVS and autofluorescent lymphocytes; red channel depicts anti-CD3 and CD19 antibodies localized to lymphocytes, including autofluorescent lymphocytes; far-red channel (pink) depicts autofluorescent lymphocytes; and blue channel depicts DAPI staining of both macrophage and lymphocyte nuclei. The merged image combines the green, red, and blue channels to show colocalization (the far-red image was omitted for clarity). The table insert illustrates four categories of intracellular lymphocyte colocalization patterns that were identified, and the corresponding arrows used to highlight examples in the images. (**B**) Quantification of the numbers of GFP-LVS-infected macrophages that contained intracellular lymphocytes at indicated time points after infection. At least 100 macrophages from two identical replicates were quantified. Values are mean +/– SD combined from three independent experiments.

In the course of assessing the effects of lymphocytes on intramacrophage trafficking of LVS, initial results prompted us to characterize the interactions of lymphocytes with uninfected macrophages as well as LVS-infected macrophages. Macrophages were either not infected or synchronously infected with GFP-LVS for 2 h, a time point at which the majority of GFP-LVS has likely escaped from the phagosome, and then co-cultured with either naïve or LVS-immune lymphocytes. These samples were fixed and stained for lymphocyte markers CD3 and CD19 (red) and DAPI (blue). Figure 4A displays representative images of these cultures 24 to 48 h after infection (or mock-infection). DAPI staining readily revealed extracellular lymphocytes, as lymphocyte nuclei were smaller and more compact than the macrophage nuclei. Round bodies, similar in size to lymphocytes, also appeared within macrophages about 24 h after addition of lymphocytes to the cultures. Some of these bodies were dimly autofluorescent in the green channel, as reflected by their diffuse appearance in the red and far-red channels (Fig. 4A, top row). These bodies were present regardless of lymphocyte priming (Fig. 4B) or macrophage infection status, indicating that they were not related to bacteria-containing vacuoles. Because these bodies were sometimes DAPI^+^, we suspected they may be phagocytosed lymphocytes that were being degraded by macrophages. Therefore, antibodies to CD3 and CD19 were used to label all T and B cells, respectively. Some, but not all, of these intracellular bodies were CD3/19 positive (Fig. 4A); these cell surface markers likely diminish during cell degradation, which may occur in phagosomes.

The intracellular bodies were grouped into four categories based on their autofluorescence, CD3/19 positivity, and DAPI staining (Fig. 4A inset chart). Because most CD3/19 is located on the surface of lymphocytes, CD3/19 staining was considered positive if located at the peripheral surface of the bodies and was not present in other channels; cells were considered autofluorescent if color was diffusely distributed throughout the cell body, in some cases with similar patterns between different channels. All four categories of intracellular bodies were found in each condition. Category A bodies (white arrowhead, Fig. 4A, middle row) displayed CD3/19 and DAPI, but were not noticeably autofluorescent in other channels, suggesting these bodies were in an early state of degradation. Category B bodies (yellow arrowhead, Fig. 4A, top row) still retained some CD3/19 markers but had become autofluorescent, which is a hallmark of dead/dying cells. Both Categories C and D bodies (large white and yellow arrows, respectively, Fig. 4A, bottom row) had lost CD3/19 expression and remained autofluorescent, and Category D bodies were not DAPI^+^, suggesting both these types of bodies were in later stages of degradation. The percentage of GFP-LVS infected-macrophages associated with presumed LVS-immune lymphocytes doubled between 24 and 48 h after infection but did not increase thereafter, and naïve lymphocytes associated with macrophages at a similar rate as LVS-immune lymphocytes (Fig. 4B).

### Splenocytes halt replication of LVS but do not impact trafficking during in vitro co-culture assays

**Figure 5:**
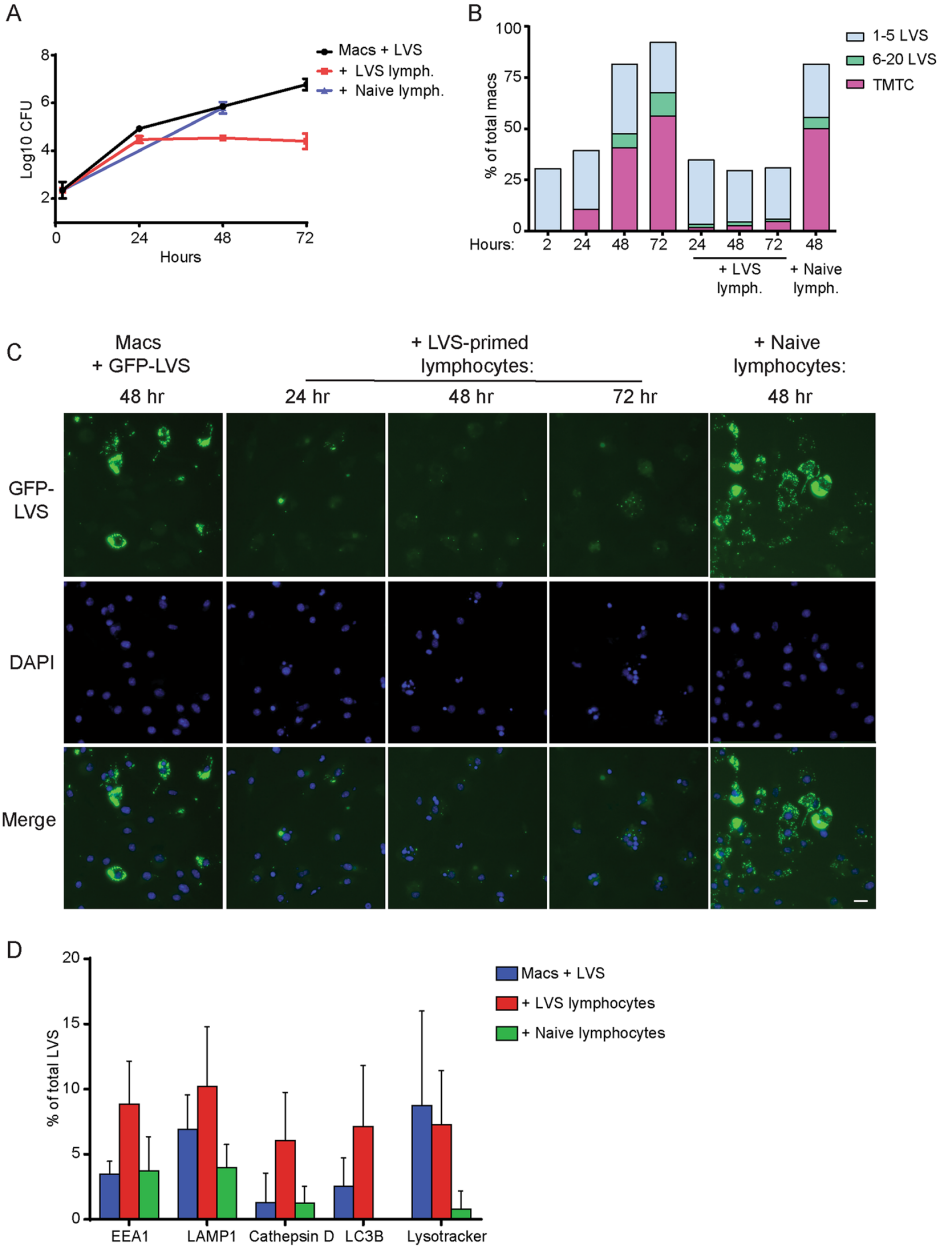
LVS-immune lymphocytes, but not naïve lymphocytes, halt intracellular replication of GFP-LVS within macrophage cytoplasm and stop intramacrophage GFP-LVS spread. GFP-LVS-infected murine BMM were co-cultured with or without splenic lymphocytes from LVS-vaccinated or naïve mice for 24–72 h, as indicated. (**A**) At the indicated time points, macrophages were washed, lysed, and plated to evaluate the recovery of intracellular bacteria. Values shown are the mean numbers of CFU/ml of viable bacteria +/– SD for triplicate samples. (**B**) Images of GFP-LVS-infected macrophages were evaluated for numbers of bacteria in each macrophage. The level of infection was categorized into four groups: macrophages containing no bacteria, 1–5 bacteria, 6–20 bacteria, and > 21 bacteria as too many to count (TMTC). At least 100 macrophages from two identical replicates were scored for each condition. (**C**) Representative images of GFP-LVS-infected BMM cultured with or without lymphocytes at the indicated time points after infection. Nucleic acid is stained with DAPI (blue). The scale bar is 50 µM. For (**A**)–(**C**), results shown are from one representative experiment of three independent experiments of similar design and outcome. (**D**) Colocalization of individual intracellular GFP-LVS bacteria 48 h after infection with the indicated markers of endosomal and autophagy pathways. At least 70 bacteria were scored from two identical replicates for each condition. Values are mean +/– SD combined from three independent experiments. No significant (*P* > 0.05) or consistent differences were found between any combination of groups.

Using results of these characterizations, we determined the fate and intracellular trafficking of LVS under the influence of immune lymphocytes. Consistent with previous studies, in the modified conditions used here to facilitate visualization, LVS-immune, but not naïve, lymphocytes greatly reduced LVS replication by 2 to 3 days after initiation of co-cultures (Fig. 5A). Numbers of LVS per macrophage and percentages of infected macrophages were quantified from fixed cell images; quantification is graphed in Fig. 5B and representative images are shown in Fig. 5C. The addition of naïve lymphocytes did not delay the replication, nor the spread, of LVS in macrophages (Fig. 5B), similar to cultures with LVS-infected macrophages only (Fig. 1B). In contrast, LVS very rarely replicated to high numbers within macrophages in co-cultures with LVS-immune lymphocytes; less than 5% of infected macrophages ever harbored more than five bacteria. Over 3 days, the percentage of infected macrophages remained stable at about 25%. These results suggest that when co-cultured with immune lymphocytes, LVS replicated little if any within macrophages, and did not spread appreciably between macrophages in the cultures (Fig. 5B, C).

We then evaluated the localization of LVS within macrophages during co-cultures. GFP-LVS-infected macrophages in the presence of naïve or LVS-immune lymphocytes 48 h after infection were stained with markers of the endosomal (EEA1 and LAMP1), lysosomal (Cathepsin D and Lysotracker), and autophagic pathways (LC3B). While there was a slight trend towards increased co-localization with four of these markers in co-cultures with LVS-immune lymphocytes, all co-localization levels were low. Differences were not significant compared to cultures with naïve lymphocytes or no lymphocytes (Fig. 5D). Instead, the majority of the LVS remained cytosolic in all conditions. Therefore, LVS-immune lymphocytes control the replication and spread of LVS in macrophages during in vitro co-cultures, but the intracellular trafficking of LVS is not substantially affected. Instead, LVS-immune lymphocytes operate by directly impacting bacterial viability within the cytoplasm of infected macrophages.

## Discussion

*Francisella tularensis* has been studied by researchers for decades, both in its own right and as a representative of intracellular pathogens generally^19,21,22,43^. The attenuated LVS strain has provided a useful experimental model for animal and in vitro models that are critical for understanding pathogenesis and immunity, as well for discovery of better treatments and vaccines. Here, we applied fixed and live-cell microscopy to visualize and test a potential T cell mechanism of bacterial growth control within the main target cells of *Francisella* infection, namely macrophages. These novel approaches demonstrated that bacteria escape from phagosomes and remain in the cytosol of infected macrophages even in the presence of LVS-immune lymphocytes that control ultimately bacterial growth. Thus, immune mechanisms that control bacterial growth function by direct influence on the replication and/or death of bacteria within the cytosol of macrophages, not by changing intracellular bacterial trafficking.

Previous in vitro studies have focused on the first 24 h after macrophage uptake of *Francisella* spp., including LVS^5–12,14–16^; none have characterized LVS infection at later time points, when some macrophages start dying and neighboring macrophages are infected. The exact mechanism of macrophage death due to *Francisella* infection has not yet been well studied at the single cell level. Previous work using population-based methods during the first 24 h of infection (e.g., upregulation of apoptotic signals, lactate dehydrogenase release, and fixed-cell imaging with propidium iodide and Annexin V) concluded that LVS-infected macrophages undergo apoptosis^13,37,38,41,42,44,45^. Pyroptotic cell death was also observed in *F. novicida* infections^39,46,47^. Apoptosis and pyroptosis are both characterized by nuclear condensation, DNA fragmentation, and surface blebbing, and distinctions between these pathways are difficult. Here, we found a dramatic increase in macrophage death after 36 h, in parallel with LVS replication across the population (Fig. 3A). We therefore considered it important to further characterize macrophage death over time, and likely to uncover a variety of mechanisms at work.

Live cell imaging, ideal for single cell analyses, confirmed that the rate of death of LVS-infected macrophages in vitro increases dramatically during the second day of infection (> 24 h). Crucially, these studies demonstrated directly at the single cell level that the death of LVS-infected macrophages is a dynamic and heterogeneous process (Fig. 3). The mechanisms of macrophage death resulting from LVS infection appeared to vary widely; these mechanisms, as well as the overall question of whether *Francisella* is inducing cell death or if the macrophage is inducing its own death, await future characterization.

The mechanism by which *Francisella* spreads between cells is also little studied to date. Recent work by Steele et al.^48,49^ showed that LVS can spread directly between cells in association with trogocytosis and when immune cells phagocytose a portion of intact cells, a processed dubbed “merocytophagy;” however, these events are relatively rare and do not account for the majority of the spread. Based on live cell imaging experiments, it appears that previously uninfected macrophages do become infected with LVS throughout the multi-day experiment in in vitro cultures, because LVS released upon macrophage death associates closely with neighboring macrophage nuclei (Supplementary Movies 1 and 2). However, it was not clear whether escaping LVS was membrane-bound in apoptotic bodies, or if bacteria escaped freely (Fig. 3B–D and Supplementary Movies 1–3). We also noted that most LVS-infected macrophages tended to either have one to five bacteria per cell or more than twenty, even at the latest time points in culture, while macrophages with intermediate burdens of six to twenty bacteria were uncommon throughout infection (Figs. 1B, 5B). Future studies will examine the implications of these observations, including whether there is a threshold of LVS replication that a macrophage can tolerate before mechanical cell death ensues.

One study to date has reported that LVS re-entered into membrane-bound vesicles, dubbed FCVs^9^, during cytosolic replication in mouse macrophages as time progresses. In order to evaluate lymphocyte effects, we first quantified the trafficking of LVS after cytosolic replication in the context of the adapted co-culture conditions applied here (Figs. 1, 2). In our hands, the vast majority of LVS remained cytosolic during infection and did not appreciably colocalize with markers of the endosomal, autophagosomal, or lysosomal pathway at any time point in either macrophages alone (Fig. 2, Supplementary Figs. 2–4) or in macrophages co-cultured with lymphocytes (Fig. 5). The methods used in this study are similar to those used in Checroun et al.^9^ but do differ slightly in how bacterial stocks were prepared and the exact strain of LVS used (i.e., independently derived isolates of GFP-LVS). Although it is possible such technical differences led to differences in intracellular trafficking, we believe this is unlikely; we also found only rare FCVs when using a different technique described earlier^9^ that is based on staining before and after vacuole membrane permeabilization (Supplementary Fig. 3) or when using staining for vacuole markers (Supplementary Fig. 4). Instead, we suggest that late-stage FCVs are rare and not a universal feature of *Francisella* cell biology.

In the course of optimizing methods to visualize events during co-culture of infected macrophages with lymphocytes, we found many macrophages containing intracellular bodies that proved to be intracellular lymphocytes (Fig. 4). Macrophages are known to engulf dead and dying cells that express phosphatidylserine, and previous studies found that some of the lymphocytes overlaid on infected macrophages died within the first 24 h of being added to the cultures^24,30,31^ (data not shown). We therefore hypothesized that these intracellular lymphocytes were dying or dead prior to being internalized. We attempted to evaluate this idea by labeling lymphocytes (prior to adding to co-cultures) with CellTracker Red, a dye that is released upon death of the cell. However, lymphocytes that died quickly released dye that appeared to then stain some of the macrophages, and phagocytosed lymphocytes leaked dye into the macrophages as lymphocytes were degraded (data not shown). These technical issues limited interpretation, but nonetheless are consistent with the possibility that macrophages preferentially phagocytose dying lymphocytes. Our present conclusion is that the differentially stained autofluorescent bodies found within macrophages (Fig. 4) were all lymphocytes in various states of degradation. In turn, we infer that results using in vitro co-cultures need to consider the potential biological and physiological consequences of macrophage engulfment of other mammalian cells. A number of other research groups use similar in vitro co-cultures to examine the mechanisms of interactions between bacterially-infected macrophages and lymphocytes^50–57^, and the conclusions drawn from such work should be interpreted accordingly in light of the results shown here. Further, the results raise the interesting possibility that these activities may routinely take place during in vivo macrophage–lymphocyte interactions, with physiological impacts that remain to be studied.

The death of infected macrophages has at least two opposing consequences to infection. Bacteria may be released to disseminate to other macrophages, but bacteria may be deprived of an important replicative niche. For bacteria contained within live macrophages, two general mechanisms of lymphocyte-induced killing of bacteria within macrophages can be postulated: intracellular bacteria may either be trafficked into toxic vacuoles such as lysosomes for degradation, or bacterial death may be induced in the cytosol. The latter possibility is represented in the best-known mechanism by which immune T cells contain intracellular pathogens, namely production of IFN-γ and/or TNF-α that engages macrophages to produce bactericidal, diffusible NO^58^. Indeed, similar to many intracellular pathogens, previous in vivo and in vitro studies clearly indicate that the cytokines TNF-α and IFN-γ are critical for control of *Francisella*, including LVS infection^25,26,59–64^. Of note, a previous study found that IFN-γ-mediated control of replication of fully virulent *F. tularensis* in murine and human macrophages was not dependent on either reactive oxygen or reactive nitrogen species; moreover, bacteria also escaped from vacuoles in IFN-γ-treated macrophages.^16^.

Here, we included treatment of infected macrophages with IFN-γ to determine the potential for intramacrophage LVS replication control in the modified culture conditions used. Similar to previous studies, IFN-γ treatment reduced but did not halt LVS replication (Fig. 1A). The magnitude of effects of IFN-γ treatment on *Francisella* intracellular replication varies with the macrophages source used, the bacterial multiplicity of infection, the time course of the study, and the IFN-γ quantity^65–69^. IFN-γ treatment may also impact trafficking of *F. tularensis* strains within macrophages^15,70^, although this has not been studied in detail. However, IFN-γ treatment never eradicates bacteria from macrophage cultures in vitro and is considerably less effective compared to bacterial growth control achieved by *Francisella*-immune lymphocytes or T cells (e.g., compare Figs. 1A with 5A). Conversely, in the in vitro co-culture approach used here, blocking IFN-γ, TNF-α, and/or NO clearly reduces bacterial growth control by *Francisella*-immune lymphocytes, but as much as 50–80% of the control remains^24–26,64,71^. Also of note, IFN-γ treatment of mouse^16,67,68^ or human^15,16,72^ macrophages has only minimal impact on the intramacrophage replication of fully virulent Type A *Francisella* strain SchuS4. Thus while IFN-γ production may be necessary during effective responses to intracellular pathogens such as *Francisella*, it is not sufficient. The goal of the present study was therefore to search for additional T cell mechanisms that provide protection against *Francisella*, such as impacting intramacrophage bacterial trafficking.

The in vitro co-culture approach was designed to mimic in vivo interactions between macrophages and lymphocytes, which are colocalized within the architecture of lymphoid organs such as spleen and lymph nodes. The relevance of this tissue culture model to in vivo infection is supported by its demonstrated value in serving as a functional correlate that predicts in vivo efficacy of *Francisella* and *M. tuberculosis* vaccines in both mice and rats^20,24,30–34^. Further, its application has previously uncovered novel mechanisms by which T cells, the dominant cell type responding to in vivo* Francisella* infection, control infection in vivo, such as IL-6 utilization and engagement of IL12Rβ2^28,29^. Here, direct visualization of macrophage–lymphocyte interactions demonstrated that LVS-immune, but not naïve, lymphocytes reduced LVS intramacrophage replication throughout the 3-day co-cultures, as shown by both CFU recovery and quantification of microscopy images (Fig. 5A). Also of note, cultured macrophages remained viable with minimal if any loss of numbers in co-cultures with LVS-immune lymphocytes (^73^ and data not shown); thus, there was no evidence for direct killing of infected macrophages (similar to cytotoxicity against virally-infected cells) as a major means of limiting bacterial growth.

Most importantly, re-routing of bacteria into autophagosomal or other degradative pathways (Fig. 5D) does not appear to be a major mechanism of growth control, because a large majority of LVS remained cytosolic in all conditions. We used whole lymphocyte populations here to allow all possible immune mechanisms to take place in co-cultures, and it is possible that more prominent contributions of re-routing are obscured by the resulting mixture of growth control activities. Future studies can therefore evaluate the impact of purified T cell subpopulations on intramacrophage trafficking. Of the small amounts of LVS that were re-routed to intracellular compartments by LVS-immune splenocytes, it was not clear whether this intracellular LVS was alive or dead, a possibility that is technically challenging to pursue. This too may be the topic of future studies, such as by using engineered bacterial reporter strains.

Collectively, the results presented here will focus future studies of T cell mechanisms to those that specifically operate within the cytoplasm of macrophages. Because many other intracellular bacteria have similar lifestyles, we expect such studies to provide new insights that will also be applicable to the larger group of these important pathogens.

## Methods

### Experimental animals

Male C57BL/6J mice were purchased from Jackson Laboratories and used when 6–12 weeks old (Bar Harbor, ME). All mice were housed in sterile microisolator cages in a barrier environment at CBER/FDA, fed autoclaved food and water ad libitum, and routinely tested for common murine pathogens by a diagnostic service provided by the Division of Veterinary Services, CBER. Within an experiment, all mice were age matched. All experiments were conducted under protocols approved by the FDA White Oak Animal Care and Use Committee and which complied with all applicable federal and state animal welfare regulations, including the U.S. Animal Welfare Act and the U.S. Public Health Service Policy on Humane Care and Use of Laboratory Animals.

### Bacteria and stocks

*F. tularensis* LVS (American Type Culture Collection 29,684), *F. tularensis* LVS expressing GFP, and *F. tularensis* expressing mCherry were used in these experiments. GFP-LVS was constructed by transfecting the pKK214GFP plasmid (a kind gift from Dr. Thomas Kawula, Washington State University) into LVS; mCherry-LVS was a kind gift from Dr. Bernard Arulanandam (University of Texas San Antonio)^74^; and LVS lacking the pdpA gene (ΔpdpA) was a kind gift from Dr. Francis Nano (University of Victoria). LVS was grown to mid-log phase in modified Mueller–Hinton (MH) broth (Difco Laboratories, Detroit, MI), as previously described^24,63,75^, harvested, and frozen in 0.5–1 ml aliquots in broth alone at − 80 °C. Each bacterial stock was quality controlled in separate experiments by determining numbers of live CFU, confirming typical colony morphologies, and confirming expected LD_50_s and times to death using adult male BALB/cByJ mice; stocks not meeting predetermined criteria were discarded^24,63^. Bacteria were periodically thawed for use and viability was quantified by plating serial dilutions on modified MH agar plates.

### Bacterial immunizations

Groups of mice were immunized by intradermal (ID) injection with 1 × 10^4^ CFU LVS, an optimal dose for maximal protection against lethal intraperitoneal LVS challenge^19,63,76^. LVS was diluted in 0.1 ml phosphate-buffer saline (PBS) (BioWhittaker, Walkersville, MD) containing < 0.01 ng of endotoxin/ml. Actual doses of inoculated bacteria were retrospectively determined by plate count. Control groups received 0.1 ml PBS ID. Splenic lymphocytes were harvested for in vitro co-culture experiments 5–10 weeks after LVS vaccination.

### Culture of bone marrow-derived macrophages and synchronized infection with *F. tularensis* LVS

Synchronized LVS infections were performed in 24-well tissue culture plates containing 12 mm round coverslips, as described previously^9^ with some modifications. BMM from naïve male C57BL/6J mice were cultured for 6 days in complete DMEM (DMEM supplemented with 10% heat-inactivated fetal bovine serum [HyClone, Logan, UT], 10 ng/ml recombinant mouse M-CSF [Biolegend], 0.2 mM l-glutamine, 10 mM HEPES buffer, 0.1 mM nonessential amino acids, 1% sodium bicarbonate, and 1% sodium pyruvate) in 100 mm dishes. After 6 days, BMMs were washed once with PBS and incubated with cold 1 mM EDTA-PBS for 20 min on ice with rocking. BMMs were harvested, resuspended in complete DMEM with M-CSF, and re-plated into 24-well tissue culture plates or 2-well chambered coverslips at a density of 1 × 10^5^ or 2 × 10^5^ macrophages/well, respectively. Re-plated BMMs were then cultured for 24 h at 37 °C in a 5% CO_2_ atmosphere. Synchronized GFP-LVS infections of macrophages at an MOI of 50:1 were performed as described^9^. Briefly, bacteria were added to wells and plates were centrifuged at 1,000 rpm for 10 min at 4 °C. Plates were warmed in a 37 °C water bath for 3 min and then placed in a 37 °C incubator for 17 min. Wells were washed 4 times with warm PBS, fresh media was added, and plates were incubated for another 40 min at 37 °C. To kill extracellular bacteria, media containing 50 µg/ml gentamicin was added for 1 h at 37 °C, after which wells were washed with PBS and fresh media without gentamicin was added. Where indicated, BMMs were incubated with recombinant mouse Interferon-γ (Millipore) at 50 ng/ml for 24 h prior to infection as well as throughout the infection culture period. At the indicated time points, intracellular bacterial loads were determined by lysing macrophages and plating intracellular bacteria on Mueller–Hinton plates; plates were incubated for 2–3 days and CFUs counted.

### Preparation of splenocytes and co-culture with infected BMMs

Single cell suspensions of splenic lymphocytes derived from naïve or vaccinated were prepared using mechanical disruption and red blood cell lysis, as previously described^23,25,30,31,71^ and added to GFP-LVS-infected macrophages using 2.5 × 10^6^ lymphocytes/well.

### Immunostaining of fixed and live cultures

Immunostaining of infected macrophages was performed as described previously^9,10,16^. Briefly, GFP-LVS infected macrophages grown on 12 mm round coverslips were washed three times with PBS, fixed with 3% paraformaldehyde at 37 °C for 20 min, and washed again three times with PBS. Coverslips were incubated for 10 min at room temperature or overnight at 4 °C in 50 mM NH_4_Cl in PBS to quench free aldehyde groups. Cells were blocked and permeabilized in 10% FBS with 0.1% saponin in PBS for 1 h; incubated in blocking buffer containing the primary antibody of interest for 40 min and washed; and then incubated with blocking buffer containing the secondary antibody for 40 min. Alternatively, cells were processed to evaluate intracellular localization using a differential permeabilization approach, as previously described^9^ with minor modifications. Briefly, BMMs were infected with GFP-LVS or mCherry-LVS and washed with buffer (110 mM potassium acetate, 20 mM Hepes, and 2 mM MgCl at pH 7.3), then treated with 50 µg/ml digitonin in KHM buffer for 1 min at room temperature. After further washing, BMMs were fixed with paraformaldehyde and 0.1% saponin, and stained again with appropriately labeled secondary antibodies. Where indicated, cells were then washed and stained with DAPI (Santa Cruz, sc-358, 1:1,000), washed, and mounted on slides in Vectashield. Primary and secondary antibodies were titrated in separate studies to optimize staining conditions. Primary antibodies used, with respective dilutions, were: rabbit polyclonal anti-EEA1 (abcam ab2900; 1:1,000); rat monoclonal anti-LAMP1 1D4B (abcam ab25245; 1:1,000); rabbit polyclonal anti-LC3B (abcam, ab48394; 1:100); rabbit monoclonal anti-Cathepsin D (abcam, ab75852; 1:100); rabbit polyclonal anti-CD3 (abcam, ab5690; 1:200); rabbit polyclonal anti-CD19 (Novus Biologicals; NBP2-15782; 1:500); goat polyclonal anti-calnexin (ab190092, 1:100); and mouse anti-*Francisella* LPS antibody FB11 (Invitrogen, 1:2,000). Secondary antibodies used were Alexa-Fluor 568- and 594-conjuguated donkey anti-rat antibodies (abcam, ab175475 and ab150156; 1:1,000 and 1:500, respectively); Alexa-Fluor 568-conjugated donkey anti-rabbit antibodies (abcam, ab 175470; 1:1,000); Alexa-Fluor 488-conjugated donkey anti-mouse antibodies (ab150105, 1:500); or Cy5-conjugated donkey anti-goat antibodies (ab6566, 1:200). ΔpdpA, which was not transfected with a reporter fluorochrome, was detected with mouse anti-*F. tularensis* LPS antibodies and donkey anti-mouse IgG-AF488 (ab150105, 1:1,000).

Live cell staining of samples to be fixed was performed in 24-well dishes on coverslips. Lysotracker Deep Red (Molecular Probes; 50 nM) and Hoescht 33342 (Molecular Probes; 1 µg/ml) were diluted in warmed complete DMEM with M-CSF but without phenol red. Cells were incubated with dyes for 30 min at 37 °C, washed three times with PBS, fixed as described above, washed again, and mounted in Vectashield (Vector Laboratories) on slides.

Live cell staining of samples for live imaging was performed in 8-well chambered coverglass (spinning-disk experiments) or 24-well dishes (Incucyte ZOOM experiments). Infected macrophages were incubated in complete DMEM with M-CSF without phenol red. DRAQ7 (abcam, ab109202; 3 µM/ml) and Hoescht 33342 (Molecular Probes; 1 µg/ml) were added directly to culture wells to visualize death of macrophages; DRAQ7 is a membrane-impermeable nucleic acid dye that only fluoresces when bound to nucleic acid in cells whose membranes have become compromised^77^. Where indicated, splenic lymphocytes were stained with Celltracker Red (Molecular Probes, C34552; 5 µM) for 30 min at 37 °C, washed, and added to macrophage cultures.

### Imaging and quantification of images

Fixed samples were observed on a Zeiss LSM710 Upright Confocal Microscope with a 20 ×, 40 ×, or 63 × objective. For each condition and primary antibody, at least two coverslips were imaged and quantified, and at least 100 bacteria were counted, using ImageJ software. An example of colocalization scoring is shown in Supplementary Fig. 1, using macrophages infected with mCherry-LVS bacteria for 4 h and then stained for detection of LAMP1. In this example, four mCherry bacteria of ten were scored as LAMP1^+^ at this time point; in general, we found the rate of LVS escape from vacuoles to be somewhat slower than previously described^9^. Live cell imaging was performed using a Zeiss CellObserver Spinning Disk Inverted Microscope in a temperature and carbon dioxide-contained chamber. Cells were imaged every 10–15 min over 24 h. For both fixed and live cell imaging studies, images were quantified and figures were assembled using ImageJ and Adobe Illustrator software.

An Incucyte ZOOM in a 37 °C, 5% CO_2_ incubator used to measure confluency, GFP-LVS, and DRAQ7 levels during GFP-LVS infection of macrophages. Cells were imaged every 4–6 h, and Incucyte’s automated image analysis software was used to determine percent confluency, total GFP-LVS area per well, and numbers of DRAQ7^+^ cells per well. Incucyte processing definition details are listed as follows:

- To automatically quantify confluency of whole well images: segmentation adjustment: 0.7; hole fill: 0.0; adjust size: 0.0; area filter: 0; processing keep out: 0.
- To automatically quantify green fluorescence: parameters: adaptive, threshold adjustment: 2.0; edge split: on; edge sensitivity: 0; clean-up: 0; filters: 0.
- To automatically quantify red fluorescence: parameters: adaptive, threshold adjustment: 1.0; edge split: on; edge sensitivity: 1; clean-up: 0; filters: 0.

## Supplementary information

Supplementary Legends.

Supplementary Movie 1.

Supplementary Movie 2.

Supplementary Movie 3.

Supplementary Figure 1.

Supplementary Figure 2.

Supplementary Figure 3.

Supplementary Figure 4.
