# Supplementary material for: Immune lymphocytes halt replication of Francisella tularensis LVS within the cytoplasm of infected macrophages
Source: Sci Rep. 2020 Jul 21;10:12023. doi: 10.1038/s41598-020-68798-2 (PMC7374111; doi:10.1038/s41598-020-68798-2)
Supplement: Supplementary file 1 — Supplementary Legends. [file 41598_2020_68798_MOESM1_ESM.docx]

**SUPPLEMENTARY DATA**

**Supplementary Movies:** Murine BMM were infected with GFP-LVS at an MOI of 50:1 (bacteria to macrophage ratio) and imaged with a spinning disk confocal microscope between 24 and 48 hours after infection.

**Supplementary Movie 1:** Time lapse images of GFP-LVS-infected macrophages. Images were taken every 15 minutes. Hoechst (blue) dye was used to label nuclei of all macrophages and DRAQ7 (pink) dye was used to label nuclei of dead cells. Movies are compressed to 10 frames/sec. Scale bar is 20 μM.

**Supplementary Movie 2:** Time lapse images of GFP-LVS-infected macrophages. Images were taken every 15 minutes. Hoechst (blue) dye was used to label nuclei of all macrophages and DRAQ7 (pink) dye was used to label nuclei of dead cells. Movies are compressed to 10 frames/sec. Scale bar is 20 μM.

**Supplementary Movie 3:** Time lapse images of GFP-LVS infected macrophages. Images were taken every 10 minutes. DIC (grey) depicts the outline of macrophages and DRAQ7 (pink) dye was used to label nuclei of dead cells. Movies are compressed to 14 frames/sec. Scale bar is 20 μM.

**Supplementary Figure 1:** LVS associates with LAMP1 at early time points after infection. Murine BMM were infected with mCherry-LVS at an MOI of 25:1 and then cultured for 4 hours. A representative image of mCherry-LVS-infected macrophages (left panel) stained for detection of LAMP1 (center panel), 63X magnification, illustrates colocalization of single mCherry-LVS bacteria (prior to replication) with LAMP1. Here, four of ten bacteria were scored as LAMP1^+^.

**Supplementary Figure 2:** Evaluation of LVS and ΔpdpA localization at early and intermediate time points. Murine BMM were cultured and then infected with either GFP-LVS ΔpdpA or at an MOI of 50:1. Colocalization of GFP-LVS or ΔpdpA bacteria with EEA1, LAMP1, cathepsin D, LC3B, and/or Lysotracker was quantified at 2 or 24 hours after infection, as illustrated in Figure 2. At least 100 bacteria were scored from two replicates in one experiment for each condition. Results are shown for colocalization of the indicated bacteria with LAMP1, which was the most associated marker for both bacteria at both time points; similar trends were seen for all other markers.

**Supplementary Figure 3:** Determination of cytoplasmic bacteria by phagosomal integrity assay. Murine BMM were cultured and then infected with GFP-LVS at an MOI of 50:1. After 1 or 24 hours, cells were processed for differential staining using a phagosomal integrity assay approach, as described in Materials and Methods. Representative images of GFP-LVS-infected (green) macrophages are shown for samples treated or not treated with digitonin, and stained for the indicated markers 1 hour or 24 hours after infection, 40X magnification. In the top row, images contain 63X magnified inserts in the lower right corner in order to illustrate single bacteria at the 1 hour time point (prior to replication). Images shown are representative of three independent experiments of similar design and outcome.

**Supplementary Figure 4:** Example of rare FCV as illustrated by LAMP1 costaining. Murine BMM were cultured and then infected with mCherry-LVS (red) at an MOI of 50:1, cultured for 24 hours, and then processed for staining of LAMP1 (green) and of nuclei (blue). A discrete mass of LVS bacteria lightly co-stained for LAMP1 (merge) suggests this LVS mass is within a LAMP1^+^ vacuole.
