# Supplementary figures and images for: Immune lymphocytes halt replication of Francisella tularensis LVS within the cytoplasm of infected macrophages
Source: Sci Rep. 2020 Jul 21;10:12023. doi: 10.1038/s41598-020-68798-2 (PMC7374111; doi:10.1038/s41598-020-68798-2)

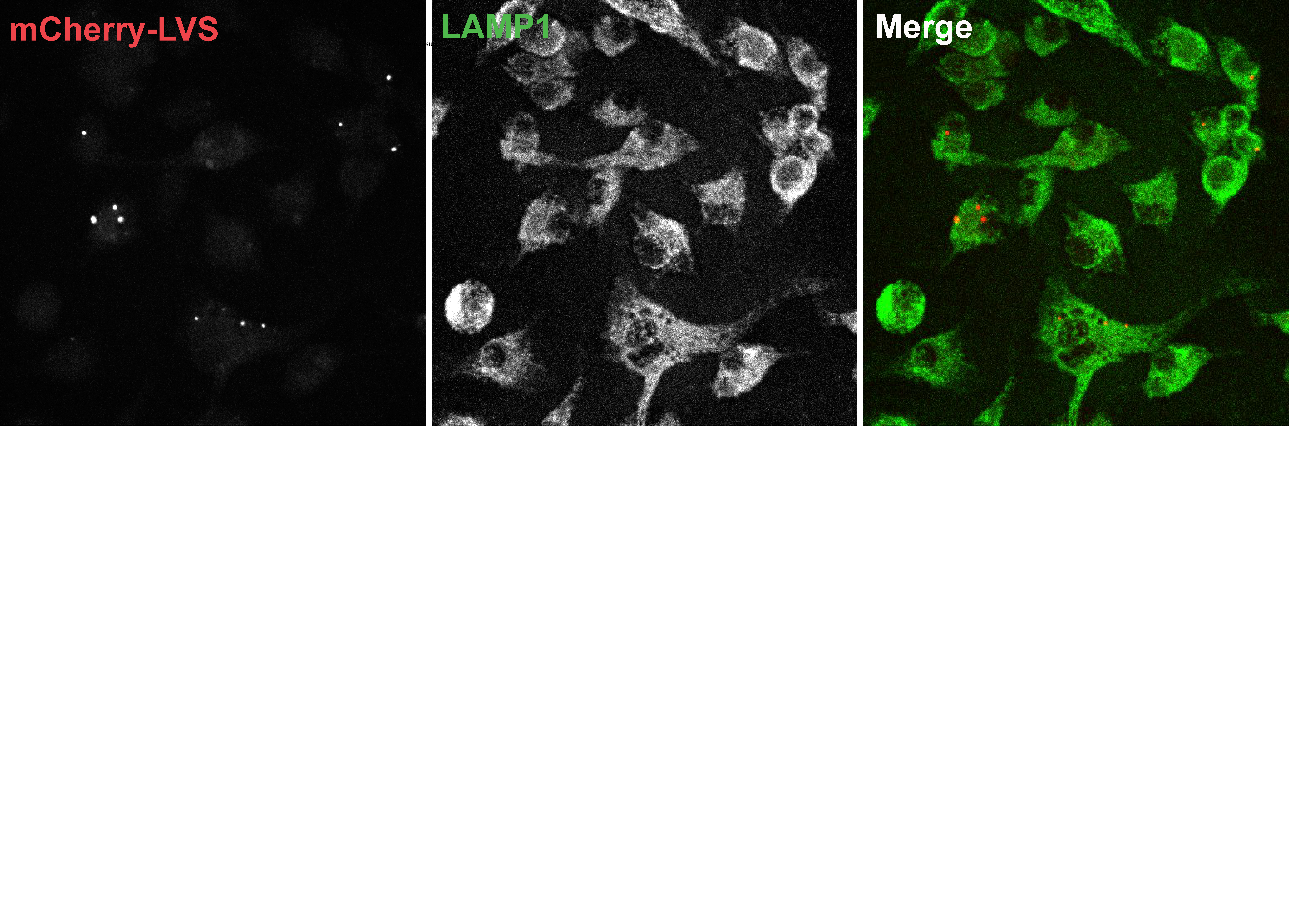

Supplement: Supplementary file 5 — Supplementary Figure 1. [file 41598_2020_68798_MOESM5_ESM.tif]

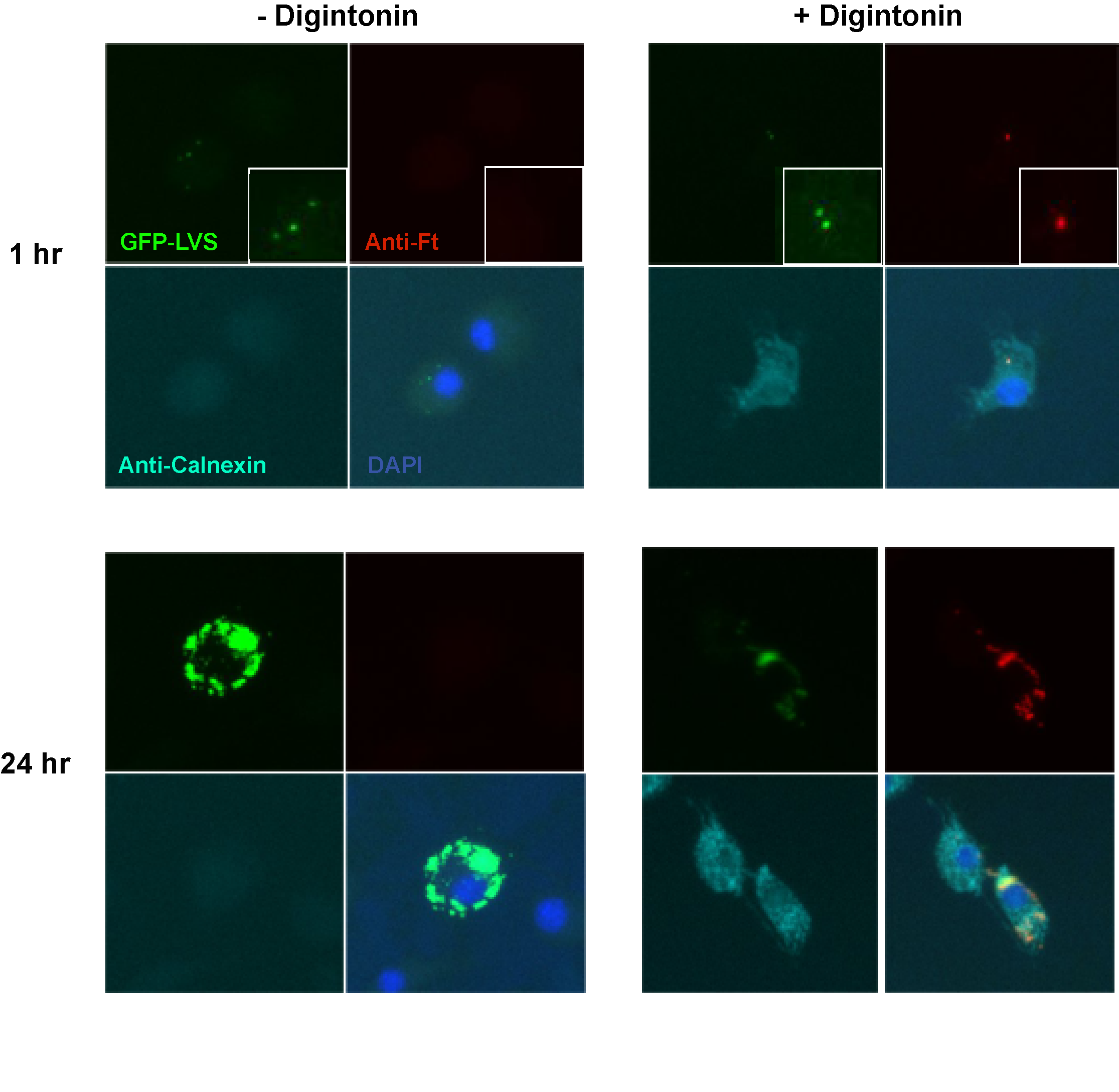

Supplement: Supplementary file 7 — Supplementary Figure 3. [file 41598_2020_68798_MOESM7_ESM.tif]

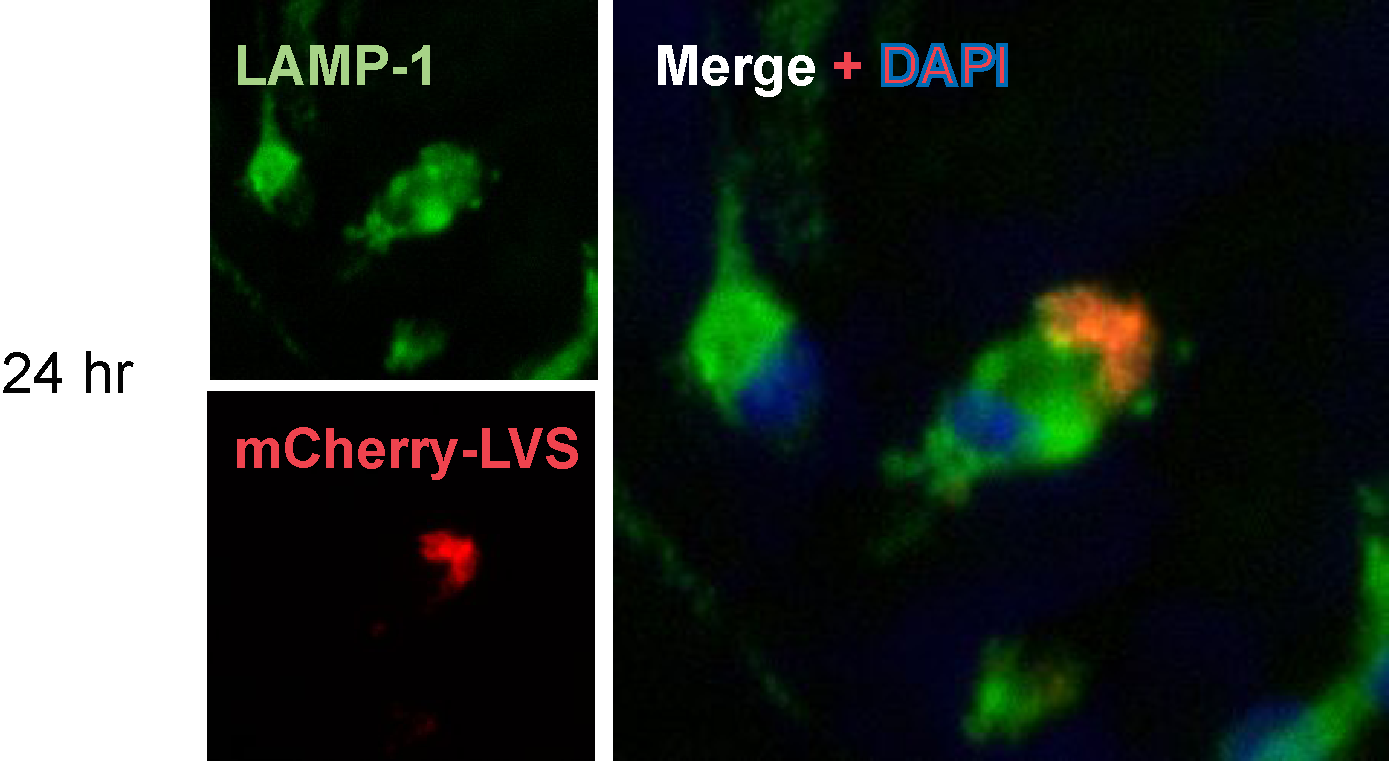

Supplement: Supplementary file 8 — Supplementary Figure 4. [file 41598_2020_68798_MOESM8_ESM.tif]
